# Uncovering ferroptosis in Parkinson’s disease via bioinformatics and machine learning, and reversed deducing potential therapeutic natural products

**DOI:** 10.3389/fgene.2023.1231707

**Published:** 2023-07-06

**Authors:** Peng Wang, Qi Chen, Zhuqian Tang, Liang Wang, Bizhen Gong, Min Li, Shaodan Li, Minghui Yang

**Affiliations:** ^1^ Postgraduate School, Medical School of Chinese PLA, Beijing, China; ^2^ Department of Traditional Chinese Medicine, The Sixth Medical Center, Chinese PLA General Hospital, Beijing, China; ^3^ School of Pharmacy, Key Laboratory for Modern Research of Traditional Chinese Medicine of Jiangsu, Nanjing University of Chinese Medicine, Nan Jing, Jiangsu, China; ^4^ Department of Gastroenterology and Hepatology, The First Medical Center, Chinese PLA General Hospital, Beijing, China

**Keywords:** Parkinson’s disease, ferroptosis, transcriptomics, machine learning, natural product, ingredient

## Abstract

**Objective:** Ferroptosis, a novel form of cell death, is closely associated with excessive iron accumulated within the substantia nigra in Parkinson’s disease (PD). Despite extensive research, the underlying molecular mechanisms driving ferroptosis in PD remain elusive. Here, we employed a bioinformatics and machine learning approach to predict the genes associated with ferroptosis in PD and investigate the interactions between natural products and their active ingredients with these genes.

**Methods:** We comprehensively analyzed differentially expressed genes (DEGs) for ferroptosis associated with PD (PDFerDEGs) by pairing 3 datasets (GSE7621, GSE20146, and GSE202665) from the NCBI GEO database and the FerrDb V2 database. A machine learning approach was then used to screen PDFerDEGs for signature genes. We mined the interacted natural product components based on screened signature genes. Finally, we mapped a network combined with ingredients and signature genes, then carried out molecular docking validation of core ingredients and targets to uncover potential therapeutic targets and ingredients for PD.

**Results:** We identified 109 PDFerDEGs that were significantly enriched in biological processes and KEGG pathways associated with ferroptosis (including iron ion homeostasis, iron ion transport and ferroptosis, etc.). We obtained 29 overlapping genes and identified 6 hub genes (TLR4, IL6, ADIPOQ, PTGS2, ATG7, and FADS2) by screening with two machine learning algorithms. Based on this, we screened 263 natural product components and subsequently mapped the “Overlapping Genes-Ingredients” network. According to the network, top 5 core active ingredients (quercetin, 17-beta-estradiol, glycerin, trans-resveratrol, and tocopherol) were molecularly docked to hub genes to reveal their potential role in the treatment of ferroptosis in PD.

**Conclusion:** Our findings suggested that PDFerDEGs are associated with ferroptosis and play a role in the progression of PD. Taken together, core ingredients (quercetin, 17-beta-estradiol, glycerin, trans-resveratrol, and tocopherol) bind well to hub genes (TLR4, IL6, ADIPOQ, PTGS2, ATG7, and FADS2), highlighting novel biomarkers for PD.

## 1 Introduction

Parkinson’s disease (PD) is a prevalent and progressive neurodegenerative disorder characterized by resting tremors, stiffness, bradykinesia, and postural instability, its incidence and prevalence rise with age (Hayes et al., 2019; Bloem et al., 2021). Epidemiological studies reveal that young-onset PD (onset age <40 years) is on the rise in China and Europe, posing a serious threat to human health (Jankovic, 2008). The pathological mechanism of PD is multifactorial, involving the death of dopaminergic neurons resulting from complex interactions between abnormal α-synuclein aggregation, mitochondrial and lysosomal dysfunction, and neuroinflammation (Bloem et al., 2021; Wang et al., 2022). Normally, dopaminergic neurons in the substantia nigra transmit dopamine to the striatum via the substantia nigra-striatum pathway, which is antagonistic to acetylcholine and participates in the regulation of motor function in the basal ganglia. In PD, the degeneration and loss of dopaminergic neurons in the substantia nigra lead to a significant reduction in dopamine levels in the striatum, resulting in relative hyperfunction of the acetylcholine system and clinical symptoms such as increased muscle tone and bradykinesia (Belarbi et al., 2020). Currently, there is no cure for PD, and available treatments only alleviate symptoms. Thus, it is crucial to identify new targets to improve the diagnosis and treatment of PD patients.

Ferroptosis is a novel form of iron-dependent and reactive oxygen species (ROS)-dependent cell death, which is distinct from apoptosis, necrosis, and autophagy. Although the exact pathogenesis of Parkinson’s disease (PD) remains elusive, the imbalance of iron homeostasis and lipid peroxidation have long been implicated as potential contributing factors in PD pathology (Yan et al., 2021). Moreover, mounting evidence strongly suggested that ferroptosis plays a significant role in the PD-related neurodegeneration. For instance, several mutations in ferroptosis genes are associated with PD, including the key regulator of ferroptosis DJ-1, autosomal recessive PD gene PARK7 and PLA2G6 (Cao et al., 2020). Additionally, the features of ferroptosis induction are highly consistent with the pathological changes observed in PD patients, including iron overload (Ayton et al., 2015), reduced GSH levels (Li et al., 1997), and reduced CoQ10 (Bersuker et al., 2019).

Transcriptomics, which involves studying the complete sequence information of RNA, is a potent tool for investigating potential gene regulation mechanisms related to complex traits and exploring the pathogenesis of PD. (Borrageiro et al., 2018; Sertbas and Ulgen, 2018). High-throughput sequencing technology, also known as next-generation sequencing (NGS), is an important tool for detecting differentially expressed genes from two or more samples, offering fast monitoring, high accuracy, and wide coverage (Borrageiro et al., 2018; Sertbas and Ulgen, 2018; Bersuker et al., 2019). In clinical settings, NGS technology is used to enhance the detection of pathogenic genes (Schilter et al., 2023; Yigit et al., 2023). For instance, high-throughput technology can be employed to identify rare variants and candidate genes linked to familial and sporadic PD (Kim J. Y. et al., 2023; Qin et al., 2023). NGS has yielded promising results in the diagnosis of neurodegenerative diseases. However, there are still several challenges, including technical limitations, high costs, lack of standardization of methods and data analysis, which require further investigation.

Natural products have been utilized for centuries for the treatment and prevention of human diseases, with a particular emphasis on plants and traditional Chinese medicines in China. Fumarate, peiminine, and aconitine alkaloids are examples of compounds extracted from natural products that have been utilized for the treatment of various ailments (Shen and Hao, 2020). Recent research has demonstrated that natural products have potential anti-Parkinson’s disease (PD) effects, attributable to their antioxidant and anti-inflammatory properties, as well as their ability to inhibit iron accumulation and maintain proteasome degradation and mitochondrial homeostasis (Solayman et al., 2017; Zhang et al., 2017). While a variety of small molecules and natural products with anti-PD activity have been identified, including flavonoid and polyphenol compounds, phenylpropanoid (coumarin) compounds, quinone compounds, saponin compounds, alkaloid compounds, and terpenoid compounds, none of them are capable of completely curing PD (Solayman et al., 2017). Therefore, it is imperative to utilize modern technologies, such as high-throughput sequencing combined with machine learning, to identify additional natural product components with anti-PD effects.

This article presents an integrated approach to analyze transcriptome data from the substantia nigra of the brain using machine learning techniques to identify potential key genes involved in the pathogenesis of PD ferroptosis. Furthermore, we aim to identify natural products that can potentially treat PD. Our study provides a valuable reference for the exploration of novel therapeutic targets and natural product-based interventions for PD.

## 2 Materials and methods

### 2.1 Microarray data retrieval

The PD dataset was obtained from the public repository NCBI GEO (http://www.ncbi.nlm.nih.gov/geo) (Barrett et al., 2013) using “Parkinson’s disease” or “Parkinson” as keywords for the search. Further screening was performed based on sequencing type (transcriptomics), species (*HOMO Sapiens*), and sample size (≥10). Finally, GSE7621, GSE20146, and GSE202665 were obtained. GSE7621 (Lesnick et al., 2007) [(HG-U133_Plus_2) Affymetrix Human Genome U133 Plus 2.0 Array] was generated on the GPL570 platform. This dataset analyzed the brain substantia nigra tissue of 9 normal samples and 16 PD samples after death. GSE20146 (Zheng et al., 2010) [(HG-U133_Plus_2) Affymetrix Human Genome U133 Plus 2.0 Array] was also generated on the GPL570 platform. This dataset consisted of 20 substantia nigra samples, including 10 PD samples and 10 control samples. GSE202665 (Diener et al., 2023) [Agilent-072363 SurePrint G3 Human GE v3 8 × 60 K Microarray 039494 (Feature Number Version)] was generated on the GPL20844 platform and consisted of 5 serum samples from PD stages 1–4, and 5 control samples.

### 2.2 Acquisition of microarray data and identification of differentially expressed genes (DEGs)

Microarray data for each GEO dataset can be obtained using R package “GEOquery”. Differential gene expression analysis to identify DEGs can be performed with R package “limma” (Ritchie et al., 2015). The identified DEGs must meet the criteria of adj. *p* < 0.05 and |log2(Fold-change)| > 1. The resulting DEGs were visualized using R packages “ggplot2” (Gustavsson et al., 2022) and “pheatmap” (Diao et al., 2018). Subsequently, we used the R package “sva” to remove batch effects, resulting in 49 control samples and 55 PD samples.

### 2.3 Identification of DEGs related to ferroptosis in Parkinson’s disease (PDFerDEGs)

FerrDb V2 (http://www.zhounan.org/ferrdb/current/) (Zhou et al., 2023) is a dedicated database for ferroptosis regulators and ferroptosis-disease associations. The database includes two categories of ferroptosis regulators: gene regulators (drivers, suppressors, markers, and unclassified regulators) and substance regulators. We downloaded the driver, suppressor, and marker regulator genes from the database and identified the DEGs and their expression levels related to ferroptosis gene regulators in the three datasets using the R package “limma” (Ritchie et al., 2015), based on sample expression levels after batch effect removal. The differentially expressed genes in PD-Ferroptosis-DEGs were filtered based on a significance level of *p* < 0.05.

### 2.4 Fitting generalized linear model and support vector machine recursive feature elimination for screening feature genes of ferroptosis

A generalized linear model was fitted using lasso regression to screen and predict feature genes of ferroptosis. The R package “glmnet” (Friedman et al., 2010), developed by the lasso regressor Trevor Hastie of Stanford University, was used. This package is characterized by fitting a range of different λ values to each previous fit, resulting in significant improvements in operational efficiency. The model loss function was set to a log-likelihood of -2-fold, and 10-fold cross-validation was performed by specifying “nfolds”.

The support vector machine (SVM) is a supervised learning algorithm used for dichotomous variables. The SVM recursive feature elimination (SVM-RFE) algorithm trains samples through a model, ranks the scores for each feature, removes the feature with the smallest score, trains the model again with the remaining features, performs the next iteration, and finally selects the optimal number of features. In this study, we used the R package “e1071” (version 1.6–8; https://cran.r-project.org/web/packages/e1071) to generate two training and testing datasets to screen the DEGs for key genes associated with ferroptosis.

To assess the performance of the selected genes, we plotted a receiver operating characteristic (ROC) curve, which shows the trade-off between specificity and sensitivity, and calculated the area under the curve (AUC) using the R package “pROC” (Robin et al., 2011). We used the intersection genes identified by both the lasso regression and SVM-RFE methods and considered genes with an AUC greater than 0.7 as accurate diagnostic markers for ferroptosis.

### 2.5 PDFerDEGs logFC functional enrichment analysis

The Gene Ontology (GO) is a widely-used tool for defining and describing gene product functions, including biological processes (BPs), cellular components (CCs), and molecular functions (MFs). The Kyoto Encyclopedia of Genes and Genomes (KEGG) is a comprehensive database that integrates genomic, chemical, and functional information. To perform GO and KEGG enrichment analysis of the intersection genes, we utilized version 3.18.1 of the R package “clusterProfiler” (Yu et al., 2012), with a *p*-value threshold of less than 0.05. Furthermore, we calculated the Z-score for each item using the R package “Goplot” (Walter et al., 2015).

### 2.6 Analysis of protein–protein interactions (PPI) and identification of hub genes

We also downloaded the string database (https://string-db.org/), which was used to filter protein-protein interactions (PPIs) between the intersection genes of the machine learning model, with the minimum required interaction score set to 0.4 and hidden connected nodes in the network. After analysis of internode relationships in the string network diagram using Perl imported into Cytoscape v3.7.1, the intersecting genes were calculated with the built-in cytoHubba plugin. After the computational results were exported, the R package “UpSetR” (Conway et al., 2017) was used to score hub genes and finally obtain the core intersection genes. Visualization of core intersecting genes was performed using the R package “pheatmap” (Diao et al., 2018).

### 2.7 Identification of intersecting genes related to active ingredients and the construction of an “Overlapping Genes-Ingredients” network

The HERB (http://herb.ac.cn/) (Fang et al., 2021) database is a natural medicine database platform that integrates high-throughput experimental data and reference mining data. The database provides functions such as browsing, searching, viewing and downloading of TCM, TCM active ingredients, target genes, diseases, high-throughput experimental and reference mining data. The obtained intersection gene targets were uploaded to this database to deduce the active ingredients by reverse, then the “Overlapping Genes-Ingredients” network was constructed. Using the computational tools integrated into Cytoscape, we calculated the topology values of individual nodes in the network, including degree, closeness, and betweenness.

### 2.8 Molecular docking

Molecular docking is a widely used computational technique in drug discovery and drug design that enables the study of the interaction and recognition of receptors and ligands. It is a theoretical simulation method for studying intermolecular interactions, predicting their binding modes and affinities (Chen et al., 2020). In this study, we employed molecular docking to investigate whether the top five ingredients, ranked by degree in the “Overlapping Genes-Ingredients” network, are capable of binding to the hub genes. The specific operation flow was as follows.

1) Protein receptor file preparation: The 3D structures of the target proteins were retrieved from the Protein Data Bank (PDB) based on the hub genes identified using the cytoHubba plugin (http://www.rcsb.org/) (Berman et al., 2000). The PDB format files of these proteins were downloaded and prepared using AutoDock Vina 1.2.0 (Eberhardt et al., 2021). This involved removing any water molecules present in the protein structures and replacing them with hydrogens. The altered protein structures were then saved in the PDBQT format.
2) The preparation of drug receptor documents involved retrieving drug molecular structures from TCMSP and PubChem (https://pubchem.ncbi.nlm.nih.gov/) databases (Ru et al., 2014; Kim S. et al., 2023). The resulting structures were processed using AutoDock Vina, which involved the addition of hydrogen atoms and detection of torsion tree root, followed by selection of the detected torsions. The final output consisted of ligand files in PDBQT format.
3) Defining docking parameters: We imported the protein receptors and small molecule receptors separately into AutoDock Vina to determine the scope of molecular docking. We set the protein acceptor as the grid’s center and adjusted the center coordinates (center X/Y/Z) and box size (size X/Y/Z) parameters to ensure that the protein was fully enclosed by the grid’s box. After this step, we exported the docking result file in Vina Config format.
4) Molecular docking and visualization were carried out using the AutoDock Vina functional module. The resulting pdqbt docking result files were imported into PyMOL (https://pymol.org/2/), which was used to visualize the docking results with the best binding energies and export them as PDB files. Detailed docking information was visualized using igplot + v.2.1 (Ye et al., 2021).

## 3 Results

### 3.1 Identification of PD DEGs and PDFerDEGs

**FIGURE 1:**
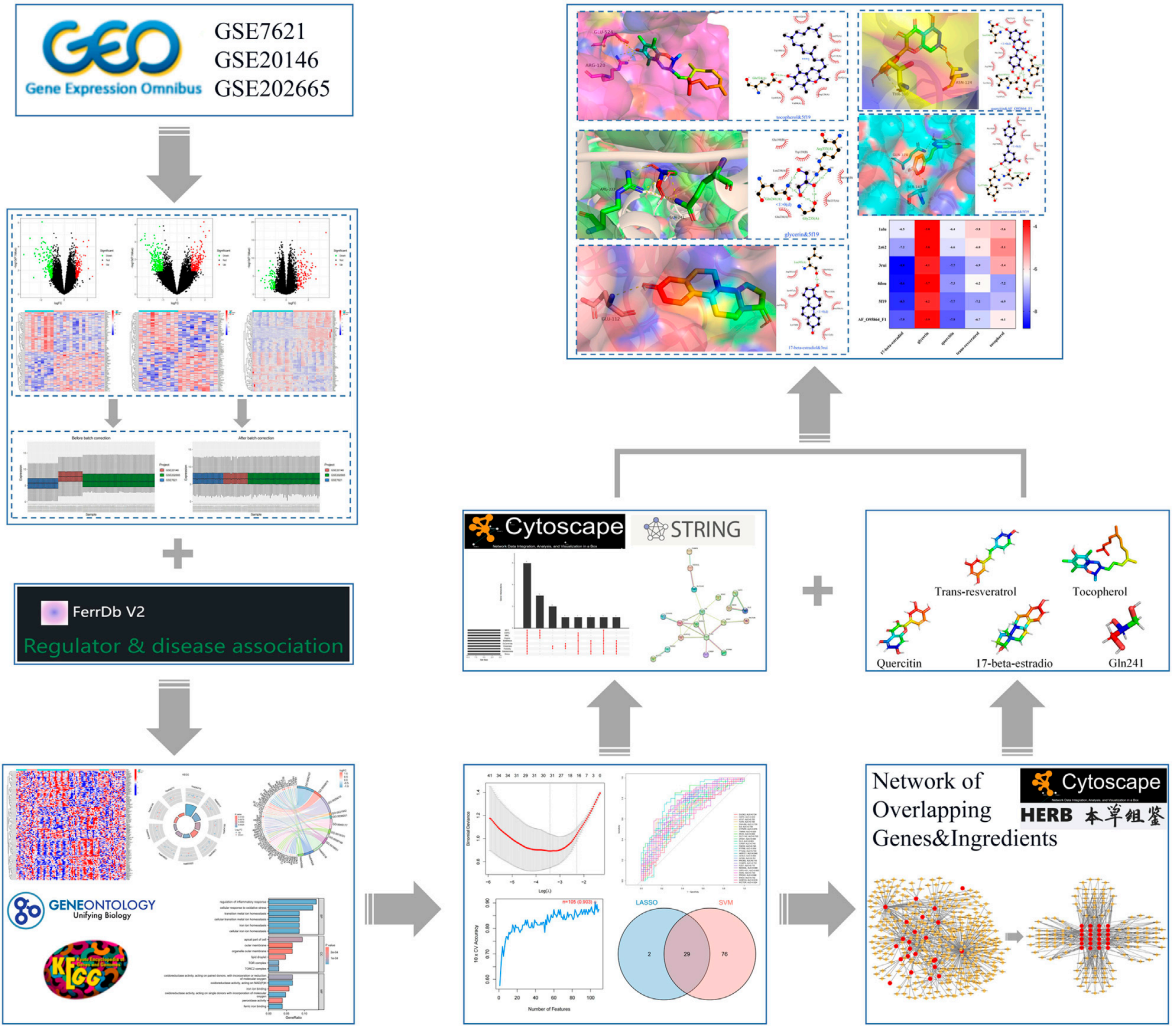
Flow chart of machine learning strategy to screen PD ferroptosis signature genes and mining potential therapeutic drugs.

**FIGURE 2:**
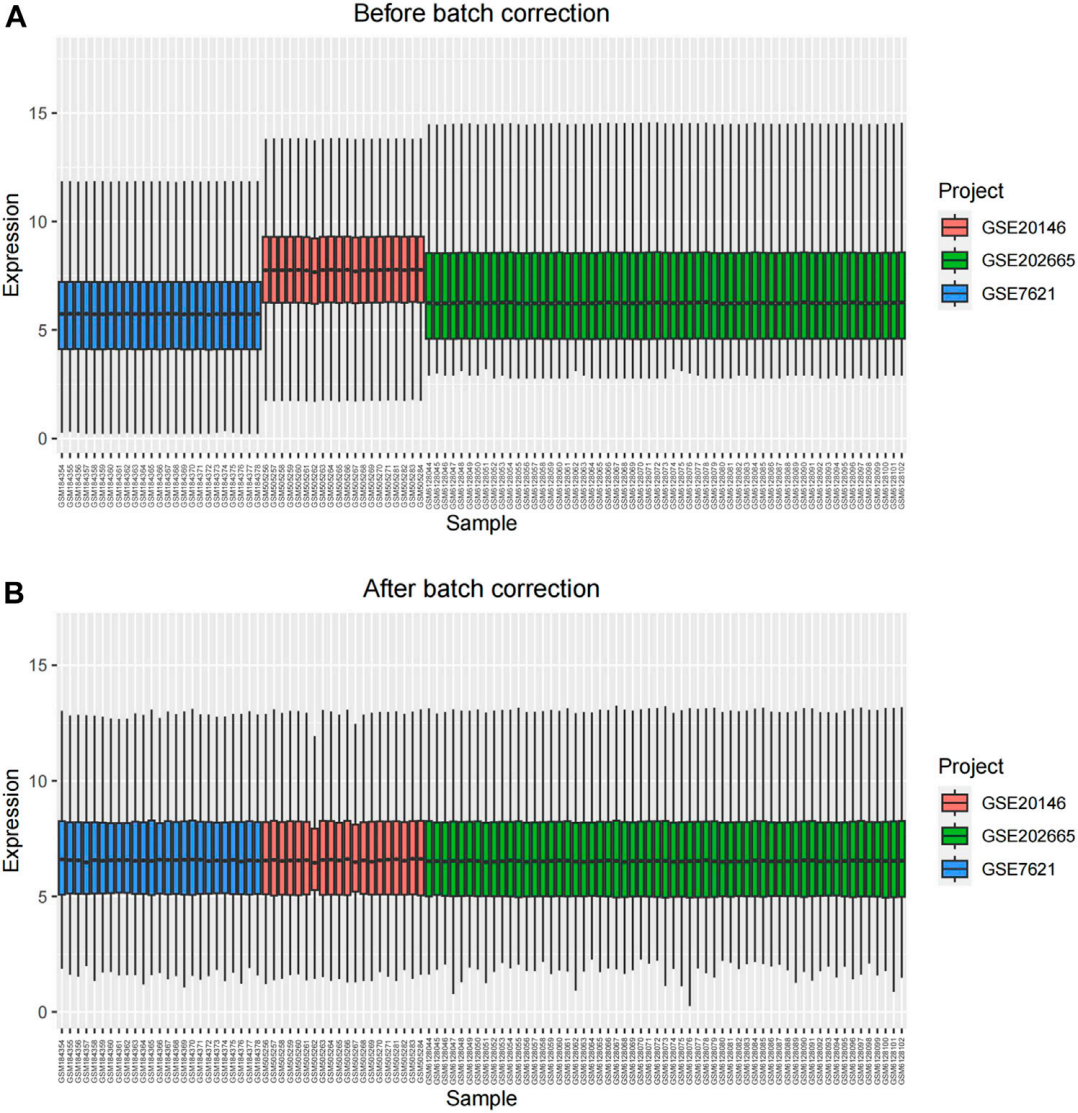
The result of eliminating batch effects. **(A)** before batch correction; **(B)** after batch correction.

**FIGURE 3:**
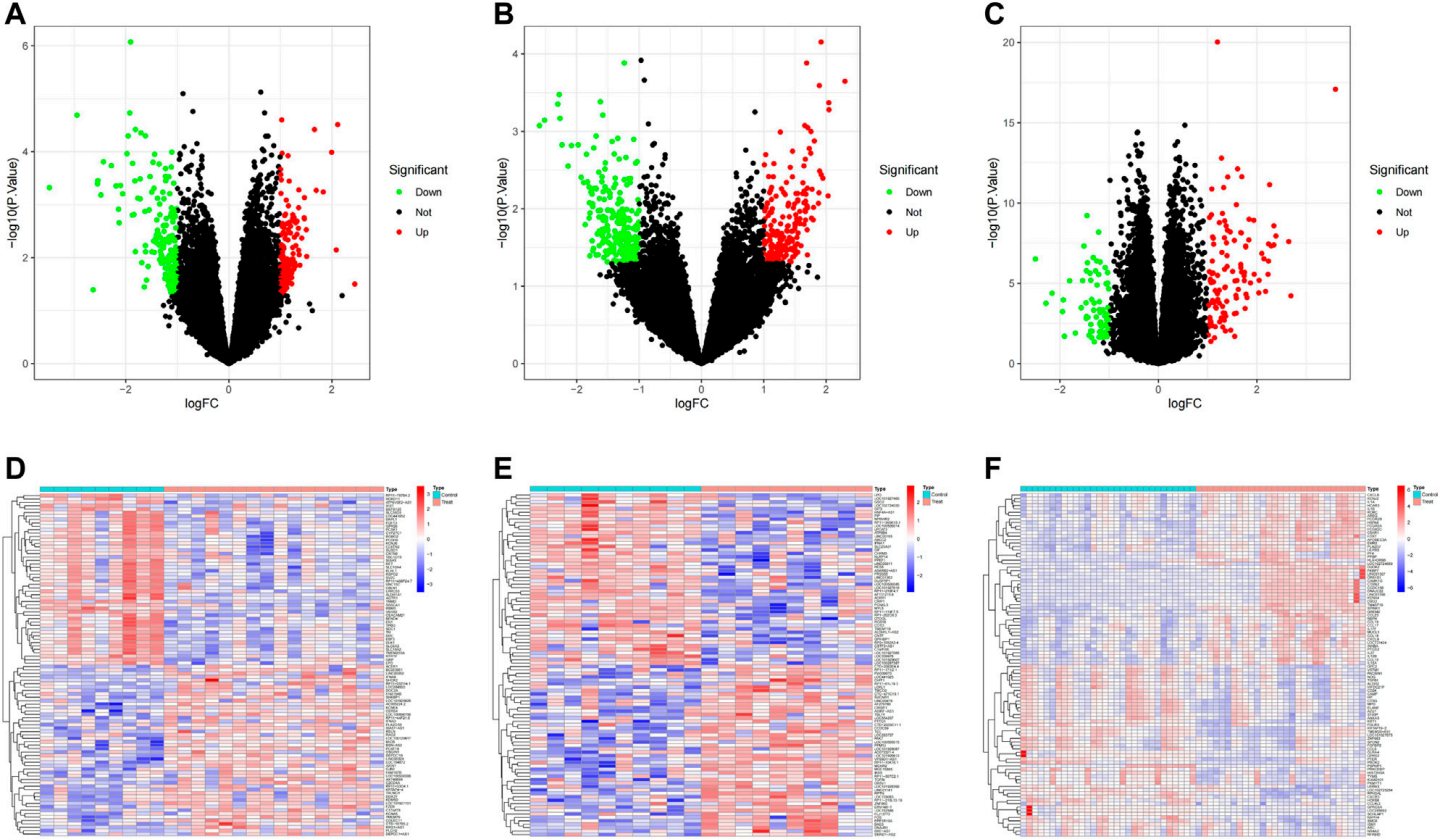
PD transcriptome dataset differential analysis. **(A–C)** volcano plot DEGs in GSE7621, GSE20146, and GSE202665; **(D–F)** Clustered heatmap of DEGs in GSE7621, GSE20146, and GSE202665.

The overall analysis strategy is shown in the flow chart in Figure 1. After eliminating the batch effect (Figure 2), three PD-related GEO datasets, GSE7621, GSE20146, and GSE202665, were obtained for analysis. There were 317 DEGs in the GSE7621 dataset, including 144 upregulated and 173 downregulated genes. There were 433 DEGs in the GSE20146 dataset, of which 197 were upregulated genes, and 236 were downregulated genes. There were 189 DEGs in the GSE202665 dataset, of which 115 were upregulated genes, and 74 were downregulated genes. The DEGs were visualized by volcano plots and heatmaps (Figures 3A–F). After eliminating batch effects across datasets, the three datasets were merged to obtain a total of 55 PD samples and 49 normal control samples (Table 1).

**TABLE 1:** The datasets used in this study.

GEO datasets	Platform	Method	Tissue	PD samples	NC samples
GSE7621	GPL570	microarray	substantia nigra	16	9
GSE20146	GPL570	microarray	substantia nigra	10	10
GSE202665	GPL20844	microarray	substantia nigra	29	30

**FIGURE 4:**
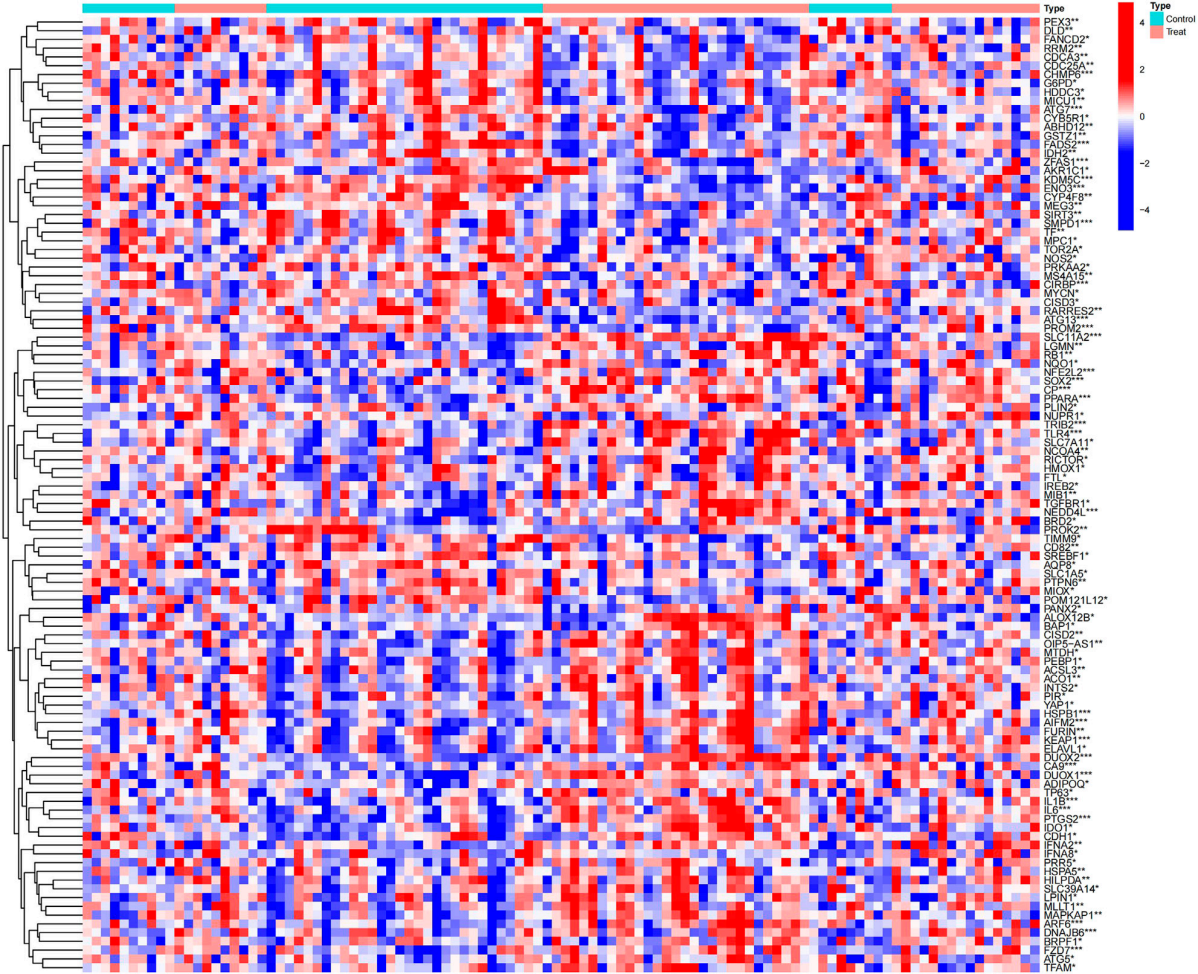
Clustered heatmap of differentially expressed ferroptosis genes in PD.

To investigate the potential link between ferroptosis and PD, we initially retrieved iron-related genes from the ferrdb V2 (http://www.zhounan.org/ferrdb/current/) database, which compiles drivers, suppressors, and markers associated with ferroptosis, resulting in a total of 728 ferroptosis-related genes. Subsequently, we identified 398 ferroptosis-associated genes that were expressed in the three datasets. Finally, as depicted in Figure 4, we obtained 109 differentially expressed ferroptosis genes in PD. Of these, 61 were upregulated genes, and 48 were downregulated genes (Supplementary Table S1).

### 3.2 Enrichment analysis of PDFerDEGs

**FIGURE 5:**
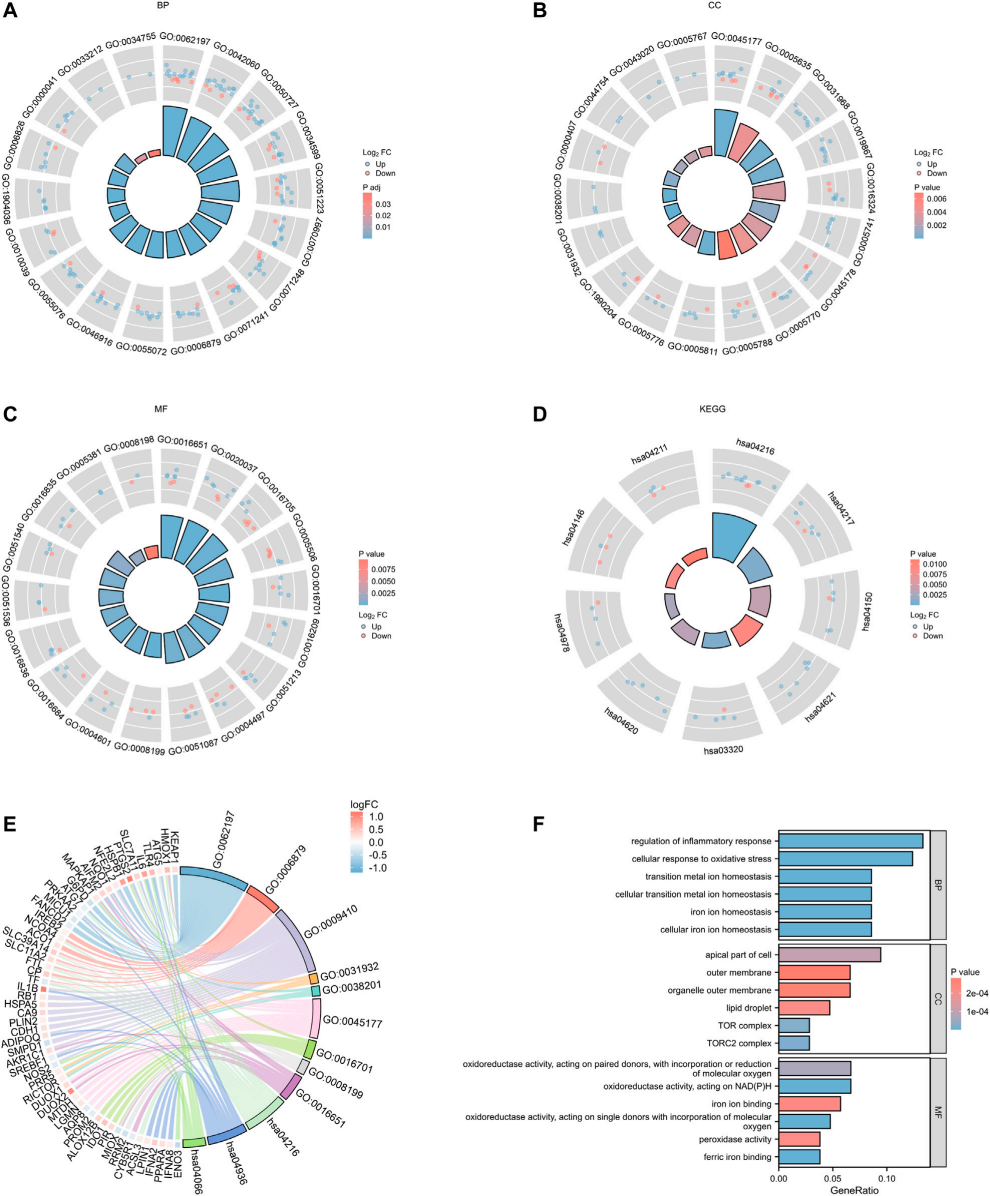
PDFerDEGs logFC functional enrichment. **(A–C)** functional enrichment of GO; **(D)** result of KEGG; **(E)** Sankey diagram combined with GO and KEGG; **(F)** overall result of GO related to ferroptosis.

Integrated with the logFC values of the 109 DEGs, enrichment analysis of the differentially expressed genes was performed. GO enrichment analysis yielded a total of 891 biological process (BP), 26 cellular component (CC) and 39 molecular function (MF) in the BP for a total of 891 biological process (BP), 26 molecular function (CC) and 39 molecular function (MF) for a total of 21 genes (IREB2, HMOX1, NCOA4, ACO1, SLC39A14, SLC11A2, FTL, CP, TF, ATG5, LGMN, PTGS2, NFE2L2, NQO1, HSPA5, CDH1, NEDD4L, G6PD, PRKAA2, SMPD1, and IREB2) involved in 11 ferroptosis processes or transport of metal ions or transport of metal ions, respectively (Table 2;Figure 5; Supplementary Table S2).In addition, 33 pathways were obtained from KEGG enrichment. Among them, 11 genes (HMOX1, ATG5, NCOA4, SLC39A14, SLC11A2, SLC7A11, ACSL3, FTL, CP, TF, and ATG7) are involved in the ferroptosis (hsa04216) pathway (Supplementary Table S3). Ferroptosis is able to participate in cell growth and death, as well as in the regulation of neurodegenerative diseases (Conrad et al., 2016).

**TABLE 2:** PDFerDEGs logFC enrichment analysis.

Items	ID	Description	*P*-value	p.adjust	Z-score
BP	GO:0006879	cellular iron ion homeostasis	1.06E-10	1.202E-07	2.333333333
BP	GO:0010038	response to metal ion	1.05464E-09	5.36885E-07	1.807392228
BP	GO:0055072	iron ion homeostasis	1.08025E-09	5.36885E-07	2.333333333
BP	GO:0046916	cellular transition metal ion homeostasis	1.60677E-08	5.15024E-06	2.333333333
BP	GO:0010039	response to iron ion	1.72711E-08	5.15024E-06	0.816496581
BP	GO:0055076	transition metal ion homeostasis	8.38227E-08	1.92276E-05	2.333333333
BP	GO:0071248	cellular response to metal ion	1.15731E-07	2.46508E-05	1.264911064
BP	GO:0006826	iron ion transport	9.31365E-07	0.000118257	1.632993162
BP	GO:0000041	transition metal ion transport	2.16779E-05	0.001154347	1.632993162
BP	GO:0033212	iron import into cell	0.001966123	0.021014265	1.414213562
BP	GO:0034755	iron ion transmembrane transport	0.00663564	0.039894113	1.414213562
CC	GO:0005741	mitochondrial outer membrane	0.000872969	0.02717116	2.449489743
CC	GO:0043020	NADPH oxidase complex	0.00252859	0.062961882	1.414213562
CC	GO:1990204	oxidoreductase complex	0.004111446	0.065790644	0
CC	GO:0005777	peroxisome	0.007245422	0.090205503	−1
MF	GO:0008199	ferric iron binding	3.19692E-07	5.21097E-05	−1
MF	GO:0016651	oxidoreductase activity, acting on NAD(P)H	6.97082E-07	7.57495E-05	1.133893419
MF	GO:0005506	iron ion binding	0.000227497	0.006742194	−0.816496581
MF	GO:0051536	iron-sulfur cluster binding	0.000581096	0.011143367	1
MF	GO:0005381	iron ion transmembrane transporter activity	0.001407298	0.020117929	1.414213562
MF	GO:0008198	ferrous iron binding	0.009577712	0.082166686	0
KEGG	hsa04216	Ferroptosis	9.80556E-14	1.95131E-11	2.110579412
KEGG	hsa03320	PPAR signaling pathway	0.000804276	0.030074846	1.341640786
KEGG	hsa04217	Necroptosis	0.000898564	0.030074846	1.133893419
KEGG	hsa04146	Peroxisome	0.008388734	0.064206081	−1

### 3.3 Machine learning models to predict ferroptosis feature genes

**FIGURE 6:**
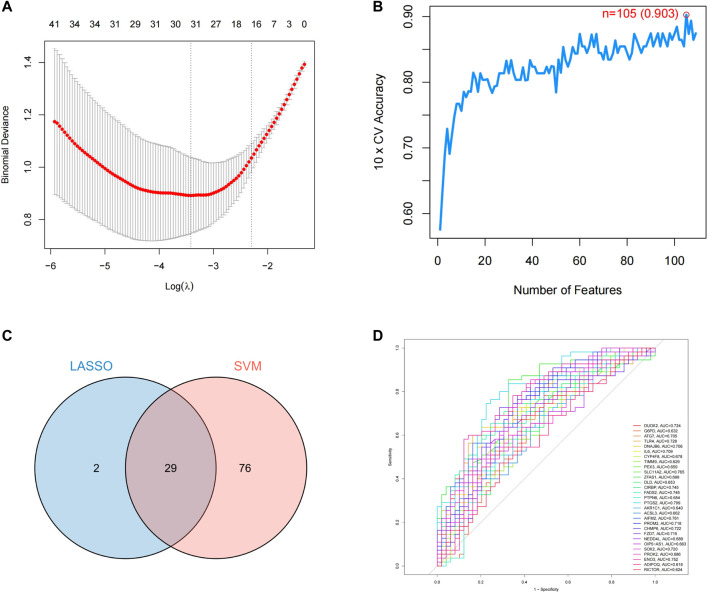
Prediction of PD ferroptosis signature genes. **(A)** lasso regression analysis; **(B)** number of features predicted by SVM-RFE; **(C)** Venn diagram of LASSO and SVM-RFE; **(D)** ROC evaluation of intersecting genes.

LASSO regression was used to analyze the resulting 109 binary discrete variables, resulting in 31 feature genes (Supplementary Table S4). Support vector machine recursive feature elimination was performed after scoring the 109 differential genes, and machine learning automatically selected the 105 feature genes with the smallest error (Supplementary Table S5). As shown in Figure 6, we screened the overlapping targets of the two prediction results by constructing a Venn diagram, resulting in a total of 29 intersecting genes. After using receiver operating characteristic (ROC) evaluation, we found that among the above intersecting genes, DUOX2, ATG7, TLR4, DNAJB6, IL6, SLC11A2, CIRBP, FADS2, PTGS2, AIFM2, PROM2, CHMP6, FZD7, SOX2, and ENO3 had an area under the curve >0.7, suggesting that the above 7 target genes have high predictive value in PD induced by the progression of ferroptosis.

### 3.4 Acquisition results of hub genes for overlapping genes and PPI network construction

**FIGURE 7:**
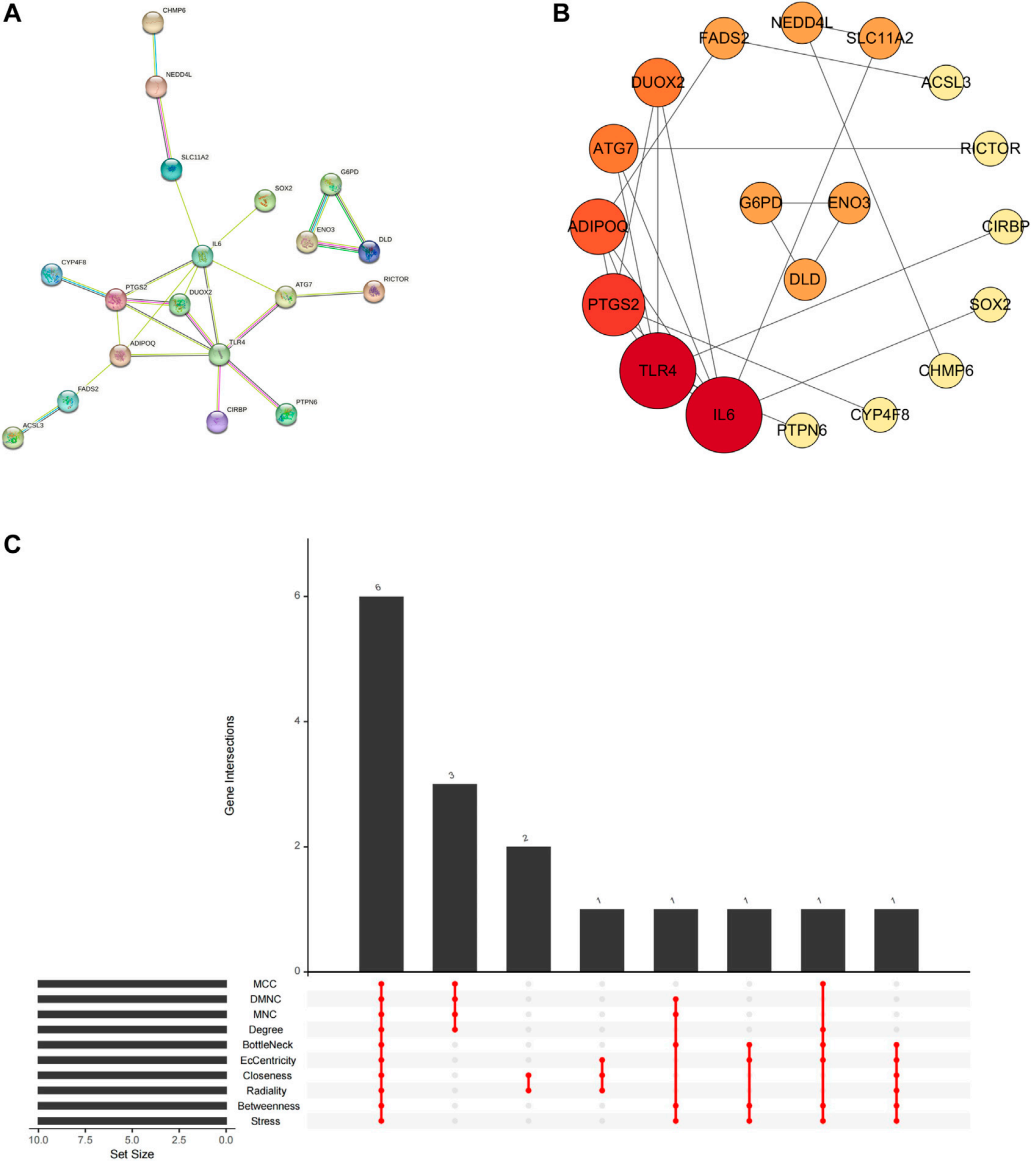
Network of PD ferroptosis signature genes. **(A)** protein-protein interaction network; **(B)** topology characteristic of each node (arranging from highest to lowest according to node degree value); **(C)** hub genes calculated by plugin cytoHubba.

In the STRING database, we first set the species as “*Homo sapiens*” and subsequently imported 29 intersecting genes to obtain the PPI network (Figure 7A), which involved 27 nodes and 24 edges. The obtained TSV files were imported into Cytoscape software (v3.7.1) for further analysis and visualization (Figure 7B). To obtain the hub genes in the intersection genes, based on the PPI network described above, we used the cytoHubba plug-in of Cytoscape, which currently contains 12 topological analysis methods. We ranked them according to MCC, DMNC, MNC, Degree, BottleNeck, EcCentricity, Closeness, Radiality, Betweenness and Stress scoring (Table 3). As shown in Figure 7C, six hub genes (TLR4, IL6, ADIPOQ, PTGS2, ATG7, and FADS2) were finally obtained.

**TABLE 3:** Hub genes of the Overlapping Gene PPI network.

Gene	MCC	DMNC	MNC	Degree	Bottle neck	Ec centricity	Closeness	Radiality	Betweenness	Stress
TLR4	10	0.37893	4	6	10	0.20833	9.41667	4.70238	60	70
IL6	10	0.37893	4	6	15	0.27778	9.66667	4.82143	96	106
ADIPOQ	7	0.46346	3	4	3	0.20833	8.41667	4.58333	48	56
PTGS2	7	0.46346	3	4	2	0.20833	8.25	4.52381	26	30
ATG7	3	0.30779	2	3	2	0.20833	7.5	4.34524	26	36
FADS2	2	0	1	2	2	0.16667	6.2	3.92857	26	30

### 3.5 Identification of overlapping genes-related compounds and construction of the “Overlapping Genes-Ingredients” network

After uploading 29 overlapping genes to HERB (http://herb.ac.cn/), we obtained the ingredients associated with Overlapping Gene. After identification, 24 genes had corresponding ingredients (Supplementary Table S6). Finally, a total of 363 ingredients related to 24 genes that were characterized after machine learning model screening were obtained (Supplementary Table S7). Although PTGS2 also had related ingredients, the number was more than three thousand, and we thought that too many ingredients were not beneficial to this study, so we did not include the ingredients related to this gene.

**FIGURE 8:**
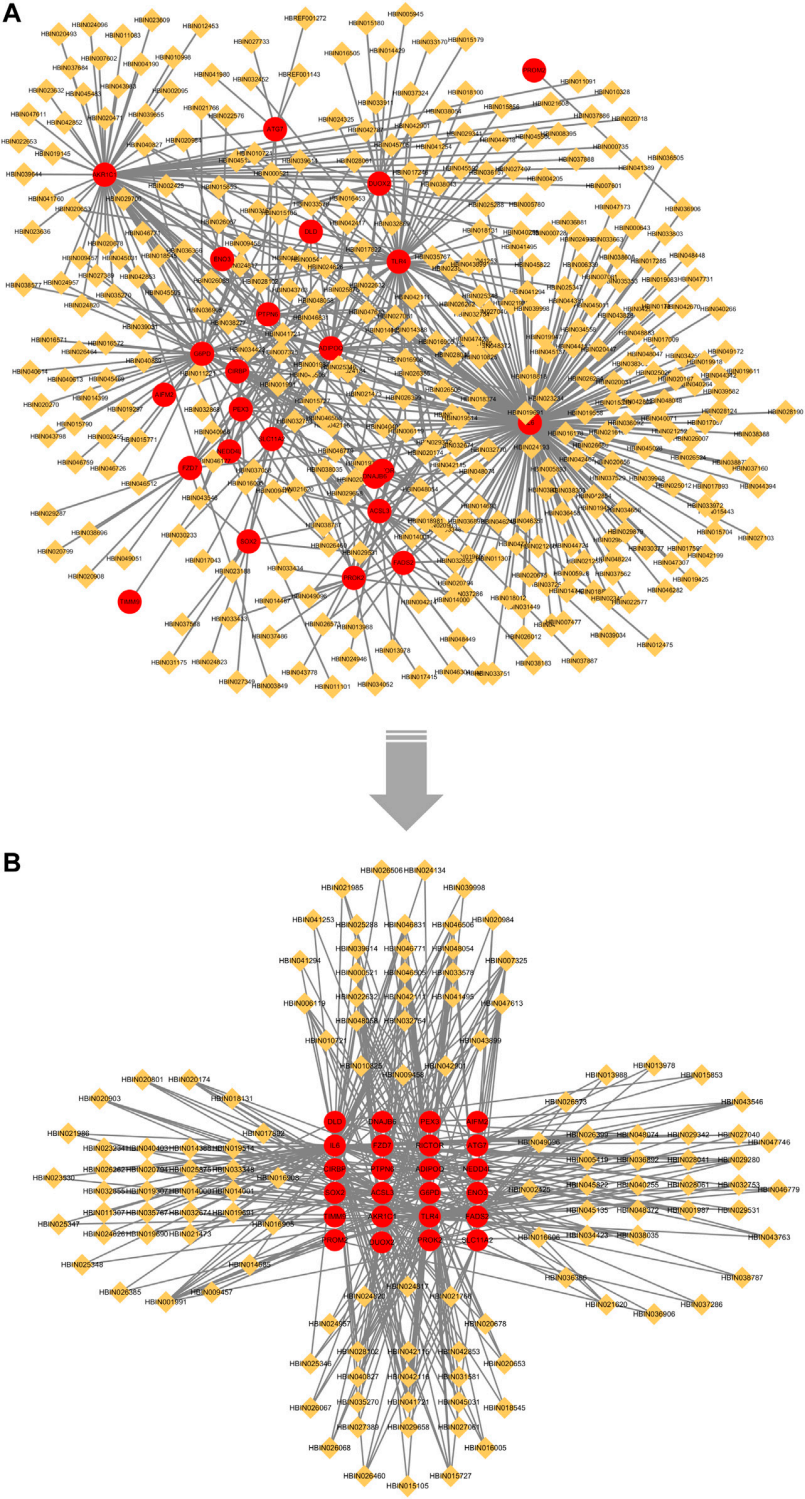
“Overlapping Genes-Ingredients” network. Red circles represent overlapping genes between LASSO and SVM-RFE; orange diamond represents active ingredient. **(A)** no median value node removed; **(B)** after removing the median value node.

We imported the correspondence between ingredients and overlapping genes into Cytoscape v3.7.1 to build the “Overlapping Genes-Ingredients” network (Figure 8A). The topological values of the nodes in the network were then calculated using its built-in calculation tool (Supplementary Table S8). We filtered ingredient nodes (Figure 8A) based on the median value and took with a median value greater than 1 (Figure 8B). Besides, the top five core ingredients were obtained according to calculation result. They were quercetin (Ingredient ID:HBIN041721), 17-beta-estradiol (Ingredient ID:HBIN001991), glycerin (Ingredient ID:HBIN028102), trans-resveratrol (Ingredient ID:HBIN046831) and tocopherol (Ingredient ID:HBIN046506) (Table 4).

**TABLE 4:** Top 5 Information of core active ingredients.

Ingredient ID	Ingredient name	Degree	Betweenness centrality	Closeness centrality
HBIN041721	quercetin	12	0.04846784	0.47201946
HBIN001991	17-beta-estradiol	11	0.04676318	0.41810345
HBIN028102	glycerin	10	0.03825909	0.44090909
HBIN046831	trans-resveratrol	10	0.03825909	0.44090909
HBIN046506	tocopherol	7	0.01554602	0.38719212

### 3.6 Molecular docking

**FIGURE 9:**
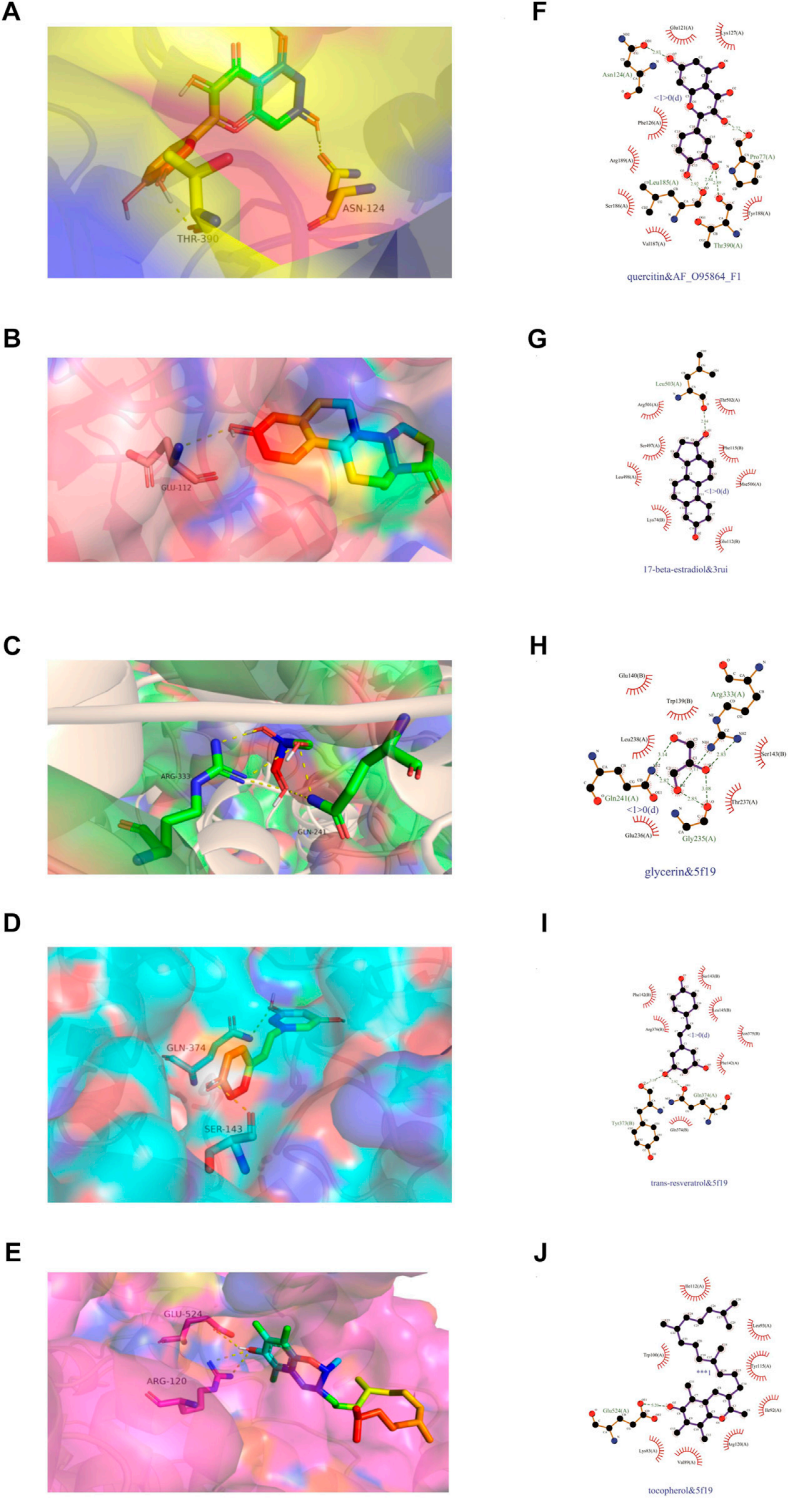
Visualization of molecular docking. **(A,F)** quercetin and FADS2, binding energy = −7.9 kca/mol; **(B,G)** 17-beta-estradiol and ATG7, binding energy = −8.8 kca/mol; **(C,H)** glycerin and PTGS2, binding energy = −4.2 kca/mol; **(D,I)** trans-resveratrol and PTGS2, binding energy = −7.2 kca/mol; **(E,J)** tocopherol and PTGS2, binding energy = −6.9 kca/mol.

We subjected the top five core ingredients of the “Overlapping Genes-Ingredients” network screening to molecular docking with the 6 hub genes TLR4 (PDB ID:2Z62), IL6 (PDB ID:1ALU), ADIPOQ (PDB ID:4DOU), PTGS2 (PDB ID:5F19), ATG7 (PDB ID:3RUI), and FADS2 (PDB ID:AF_AFO95864F1) calculated by cytoHubba. The docking results of compounds with proteins are shown in Figures 9A–E. Figures 9F–J shows the amino acid residue information versus hydrogen bonding distance for the docking of each compound to proteins.

As a result (Table 5), the top five ingredients could all interact with the six hub genes. Quercitin has the smallest binding energy to FADS2. Figure 9F shows that quercetin formed 1 hydrogen bond with Asn124, Pro77 and Thr390, and 2 hydrogen bonds with Leu185 in FADS2. 17 beta estradio has the smallest binding energy to Atg7. Figure 9G shows that 17 beta estradio formed 1 hydrogen bond with Leu503 in ATG7. Glycerin has the smallest binding energy to PTGS2. Figure 9H shows that glycerin formed 2 hydrogen bonds with Gln241, Gly235 and Arg333 in PTGS2. Trans resveratrol has the smallest binding energy to PTGS2. Figure 9I shows that trans resveratrol formed 1 hydrogen bond with Tyr373 and Gln374 in PTGS2. Tocopherol had the smallest binding energy to ADIPOQ but could not exhibit its hydrogen bond, so we selected the docking results of PTGS2 for visualization. Figure 9J shows that tocopherol formed 1 hydrogen bond with Glu524 in PTGS2.

**TABLE 5:** Molecular docking binding energy.

Ligand	Binding energy (kcal/mol)					
	1alu	2z62	3rui	4dou	5f19	AF_O95864_F1
17-beta-estradiol	−6.5	−7.2	−8.8	−8.6	−8.3	−7.9
glycerin	−3.8	−3.8	−4.1	−3.7	−4.2	−3.9
quercitin	−6.4	−6.6	−7.7	−7.3	−7.7	−7.9
trans-resveratrol	−5.8	−6	−6.9	−6.2	−7.2	−6.7
tocopherol	−5.6	−5.1	−5.4	−7.2	−6.9	−6.1

**FIGURE 10:**
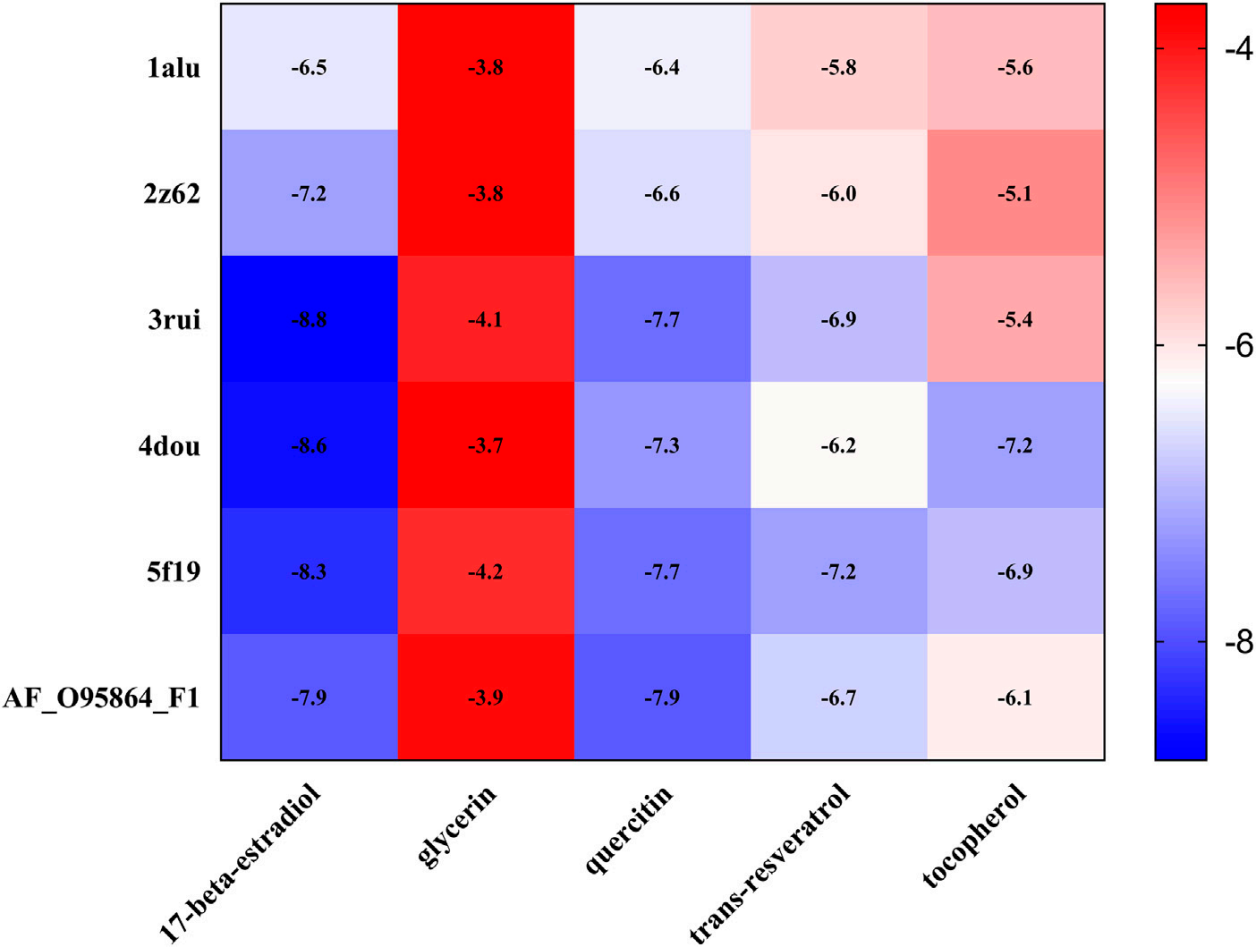
Heatmap of molecular docking binding energy between core ingredients and hub genes.

The binding energy results of the docking between each compound and hub genes are shown as a heatmap (Figure 10), and it can be seen that the binding energy ranged from −3.7 kca/mol to −11.5 kca/mol, indicating that these compounds may be potential therapeutic ingredients for hub genes.

## 4 Discussion

High-throughput can help us better understand the complexity and diversity of biological systems, gives us a clearer understanding of gene expression profiles in living organisms. Machine learning, a branch of artificial intelligence, is a process of learning from experience. It can train a model with a large amount of data, which enables the prediction and classification of unknown data. Currently, although there are individual bioinformatics reports and studies on ferroptosis in PD, the development of compound prediction based on machine learning has not been reported (Moni et al., 2019; Falchetti et al., 2020; Jian et al., 2022). This study is the first to integrate bioinformatics analysis methods, machine learning model prediction classification, and data mining-based inverse derivation of active ingredients, screen core compounds of feature gene action derived from machine learning predictions, and validate using molecular docking.

In this study, we first integrated the transcriptome datasets of three human brain substantia nigra tissues from the GEO database, combined with the targets included in the public ferroptosis database, bioinformatically analyzed the expression of ferroptosis-related genes in the three datasets, and finally obtained 103 genes that were differentially regulated in ferroptosis. Ferroptosis DEGs functional enrichment found that 11 BP items were directly related to ferroptosis or metal ion transport processes such as cellular iron ion homeostasis, iron ion transport, and transport metal ion transport. In addition, KEGG enrichment was supported by the ferroptosis (hsa04216) pathway. This illustrates that ferroptosis may contribute to the onset and progression of PD. Subsequently, to further predict the ferroptosis feature genes for PD, we predicted and classified 109 ferroptosis differential genes using two machine learning models, Lasso regression and SVM-RFE. The intersection of the genes obtained from the two prediction models was used to obtain 29 ferroptosis feature genes after machine learning. A total of 363 related compounds were derived from feature genes. According to the correspondence between compounds and targets, we constructed the “Overlapping Genes-Ingredients” network, and after calculating the values of node topology, the top five core ingredients were obtained, namely, quercetin, 17 beta estradiol, glycerin, trans resveratrol, and tocopherol. In addition, PPI network node topology analysis revealed 6 hub genes among the 29 characterized genes, i.e., TLR4, IL6, ADIPOQ, PTGS2, Atg7, and FADS2.

A total of 109 ferroptosis DEGs were used for functional enrichment analysis, including BP, CC, MP, and KEGG. Cellular iron ion homeostasis, iron ion transport, and cellular response to oxidative stress were significantly enriched in biological processes. Cellular iron homeostasis is tightly regulated to maximize iron supply in times of cellular iron deficiency and to limit iron supply and facilitate storage when cellular iron is adequate. Cellular iron ion homeostasis (Anderson and Frazer, 2017) refers to the process of maintaining the internal steady state of iron ions at the cellular level. Iron ions, as one of the metals whose homeostasis is essential for the physiological function of the brain, perturbed homeostasis, may be responsible for causing specific local cell death. It has been found that substantia nigra pars compacta iron levels are significantly elevated in PD disease progression. Elevated iron concentrations predispose the brain to oxidative stress. Iron ion transport (Anderson and Frazer, 2017) refers to the directed movement of iron ions into and out of the cell either within or between cells via transporters or other transport vehicles. Metal ion transporters are involved in maintaining the required levels of various metal ions in the cell (Bowers and Srai, 2018). Thus, increased iron levels in the substantia nigra pars compacta are associated with the transport of ferric ions. Cellular response to oxidative stress refers to any process that changes cell state or activity due to oxidative stress, usually caused by exposure to high levels of reactive oxygen species, such as superoxide anions, hydrogen peroxide (H_2_O_2_), and hydroxyl radicals. Iron in nigral neurons is primarily bound to the biopolymer neuromelanin (NM) (Zecca et al., 2001), and this binding may provide a degree of antioxidant defense (Belaidi and Bush, 2016). However, an imbalance in iron homeostasis, such that free iron exerts toxic effects, catalyzes the Fenton reaction and produces damaging reactive free radicals, causing oxidative stress and, ultimately, dopaminergic neurons (Ganz, 2003). The above three biological processes suggested that the enriched genes were associated with the imbalance of intracellular iron homeostasis in dopaminergic neurons and caused oxidative stress, which ultimately led to neuronal cell damage.

Pathways related to ferroptosis were significantly enriched in KEGG pathway analysis, including ferroptosis, PPAR signaling pathway, peroxisome, necroptosis and Toll-like receptor signaling pathway. Ferroptosis is considered a novel form of regulated cell death resulting from severe lipid peroxidation and depends on reactive oxygen species (ROS) production and iron overload (Dixon et al., 2012; Do Van et al., 2016). As nuclear receptors, PPAR signaling pathways (PPARs) are expressed in neurons and astrocytes of the central nervous system, and studies have confirmed that PPARs can play a neuroprotective role against oxidative damage, apoptosis, and neuroinflammation during the progression of PD (Heneka and Landreth, 2007; Iranpour et al., 2016). The peroxisome is an important organelle in this signaling pathway, and its biogenesis starts from the early peroxidase pex3, which can participate in key processes such as free radical detoxification (Wanders and Waterham, 2006). Increased levels of free iron lead to the formation of reactive hydroxyl radicals, resulting in an oxidative stress response that accelerates dopaminergic neuronal death, and pex3 can antagonize neuronal injury by free radical detoxification (Ganz, 2003). Necroptosis, a form of programmed necrosis, is crucial in nervous system inflammation and can be caused by Toll-like receptors (Zhang et al., 2019). Inflammatory responses mediated by Toll-like receptor signaling pathways can result from engagement of the TLR4 (included among the significantly enriched pathways) with associated ligands, allowing inflammatory factors to be released and causing inflammation (Kagan et al., 2008; Netea et al., 2009). In addition, proinflammatory cytokines released by microglia exacerbate neuronal iron deposition, increase neurologic iron overload, and ultimately exacerbate ferroptosis in dopaminergic neurons (Healy et al., 2016; Dachert et al., 2020). These findings demonstrate that 109 DEGs can be involved in ferroptosis-related processes, demonstrating the potential of these target genes as research targets.

The hub genes obtained from machine learning postscreening provide references for further exploring the core target genes of PD ferroptosis pathogenesis progression. TLR4 is one of the key targets of inflammasomes triggered by the Toll-like receptor signaling pathway in the KEGG pathway, which not only participates in neuroinflammation but also induces necroptosis (Netea et al., 2009). Misfolded α-synuclein activates microglia to release IL6 and promote intracellular iron accumulation in neurons (Sterling et al., 2022). Furthermore, it was found that when organotypic hippocampal cultures were exposed to ferrous ammonium sulfate, ferric ammonium citrate (FAC), or ferrocene, microglial activation became evident, as evidenced by increased ferritin expression in microglia, as well as IL6 proinflammatory factor release (Wang et al., 2013; Healy et al., 2016). IL6 is involved in the inflammatory response and the progression of iron accumulation in neuronal cells. ADIPOQ is an adipokine that acts as a metabolic controller involved in the metabolism of fatty acids (Yamauchi et al., 2002) and has antioxidant and anti-inflammatory effects (Liu et al., 2015). The activity of ADIPOQ involved in metabolism is induced by its three receptors, namely, T-cadherin, ADIPOQR1 and ADIPOQR2, but also by PPAR γ- α of the signaling cascade (Thundyil et al., 2012). ADIPOQR1 and ADIPOQR2 have been reported to be expressed in primary human astrocytes (Hillenbrand et al., 2012), which can mediate proinflammatory signaling in astrocytes by elevating IL6 (Wan et al., 2014). Investigation of this coding gene in PD has not been reported, which deserves our attention. Prostaglandin-endoperoxide synthase (PTGS) is also known as cyclooxygenase (Hla and Neilson, 1992). PTGS2 (COX2), a neuroinflammatory marker (NMS), plays a role in the occurrence of neurodegenerative diseases (Hoozemans et al., 2008). COX2 is induced in cells undergoing ferroptosis (Li et al., 2017). Therefore, COX2 can be considered one of the crucial markers of ferroptosis (Zuo et al., 2021). The ATG7 gene encodes an E1-like activating enzyme implicated in the regulation of organismal mitophagy (Barrientos-Riosalido et al., 2023). Studies have shown that silencing ATG7 expression can slow the proinflammatory response of microglia, thereby slowing the progression of inflammation-mediated neurotoxicity during PD (Burguillos et al., 2011; Friess et al., 2021). FADS2 is a fatty acid desaturase (FADS) gene family member. Its long-chain polyunsaturated fatty acids (PUFAs), which are involved in biosynthesis, are most highly expressed in the brain and play an essential role in inflammatory processes (Park et al., 2011; Fagerberg et al., 2014). Notably, the involvement of FADS2 in the progression of PD pathogenesis has not been reported, and this gene provides a potential target for investigation in our future studies.

Molecular docking results showed that quercetin (QCT) and 17 beta estradiol (E2) had high binding energies to all the hub genes (Table 5). QCT is a flavonoid with anti-inflammatory, antioxidant and anti-ferritin functions (Boots et al., 2008; Tang et al., 2020). In addition, it ameliorates mitochondrial dysfunction, one of the hallmarks of ferroptosis (Dixon et al., 2012; de Oliveira et al., 2016). The mechanism of QCT is to protect neurons by inhibiting microglial activation (Han et al., 2021). E2 has protective effects against many neurodegenerative diseases and mediates its effects through dopamine receptors (Brann et al., 2007; Varmazyar et al., 2019). A study has shown that E2 can activate autophagy by regulating the expression of UKL1, thereby preventing the α-abnormal accumulation of synuclein and exerting a neuronal protective effect (Varmazyar et al., 2019). This suggests that QCT and E2 may serve as potent ingredients to explore the regulatory impact on the pathogenesis of ferroptosis in PD.

Despite the identification of ferroptosis feature genes and hub genes related to PD by machine learning model screening, this study has some limitations that need to be addressed. The lack of *in vivo* and *in vitro* experiments to validate the regulatory relationship between the feature genes derived from the screen and natural products against ferroptosis in PD is a noteworthy limitation. Additionally, the potential therapeutic agents identified in this study require further comprehensive and detailed experimental analysis. Nevertheless, our study provides valuable insights into the exploration of PD ferroptosis feature genes and therapeutic agents by combining transcriptome data with the ferroptosis database. The machine learning model prediction was used to identify the feature genes, which were then combined with natural products of traditional Chinese medicine to explore potential therapeutic agents. These findings might pave the way for future research in this area.

## 5 Conclusion

As neurodegenerative disease, PD has a slowly progressive course and positive correlation between incidence and age. It has attracted increasing attention with the arrival of the global aging society. Ferroptosis, a regulated iron-dependent cell death pathway involving the fatal accumulation of lipid peroxides, shares several features with the pathophysiology of PD. Machine learning is becoming more widely applied to predicting and deciding new data. In the present study, we analyzed 3 integrated transcriptome datasets and found 109 DEGs related to ferroptosis in PD, and the related pathways were identified by GO and KEGG analyses. Subsequently, 29 feature genes were predicted after screening by machine learning. PPI network topology value analysis of feature genes found that TLR4, IL6, ADIPOQ, PTGS2, Atg7, and FADS2 may be hub genes in the progression of PD ferroptosis. Notably, we performed data mining based on reverse derivation for natural products related to the 29 feature genes and clarified the top 5 core active ingredients according to the “Overlapping Genes-Ingredients” network, followed by molecular docking for validation, suggesting that quercetin, 17-beta-estradiol, glycerin, trans-resveratrol, and tocopherol might serve as potential therapeutic agents against the ferroptosis process of PD.

## Data Availability

The datasets presented in this study can be found in online repositories. The names of the repository/repositories and accession number(s) can be found in the article/Supplementary Material.
